# Valorization of egg-white byproducts into biodegradable hydrogels: Processing optimization and functional properties

**DOI:** 10.1016/j.psj.2026.106472

**Published:** 2026-01-18

**Authors:** Yu-Shan Chang, Jr-Wei Chen, Sheng-Yao Wang, Yi-Chen Chen

**Affiliations:** a Department of Animal Science and Technology, National Taiwan University, Taipei City 106037, Taiwan; b Department of Animal Industry, Ministry of Agriculture, Executive Yuan, Taipei City 100212, Taiwan; c Master Program in Global Agriculture Technology and Genomic Science, International College, National Taiwan University, Taipei City 106319, Taiwan; d Experimental Farm, Bioresources and Agriculture, National Taiwan University, Taipei City 106032, Taiwan

**Keywords:** Abnormal eggs, Superabsorbent hydrogel, Absorbency, Integrity, Biodegradability

## Abstract

The growing accumulation of inedible abnormal eggs and environmental concerns regarding petroleum-based superabsorbent polymers (SAPs) in food packaging underscore the need for sustainable, food-derived alternatives. In this study, biodegradable egg-white hydrogels were developed through controlled acylation and crosslinking to enhance absorbency and structural stability. Processing optimization of lyophilization, heating, and grinding reduced production time from 4 to 1.5 days and significantly improved water uptake (*p* < 0.05). Among protein concentrations tested, a 4% (w/w) egg-white solution achieved the optimal balance between gel stability and water uptake, exhibiting significantly higher swelling capacity than lower or higher concentrations (*p* < 0.05). Chemical modification using succinic anhydride (SA) or ethylenediaminetetraacetic dianhydride (EDTAD), combined with glycerol (G) or N,N′-methylenebisacrylamide (MBA), produced five hydrogel formulations. Although SG (SA + *G*) and EG (EDTAD + *G*) showed lower initial swelling capacity than EM (EDTAD + MBA), both SG and EG exhibited significantly higher water-holding and reswelling capacities compared with EM (*p* < 0.05). Rheological analysis indicated that SG possessed the highest storage modulus (G′) and lowest tan δ, characteristic of a predominantly elastic network, whereas EG displayed increased viscosity and plasticity due to enhanced hydrophilicity. In contrast, MBA-crosslinked hydrogels showed weaker network formation and limited swelling performance. Soil burial tests demonstrated that SG and EG hydrogels degraded more rapidly than commercial water beads and promoted greater microbial growth within 7 days (*p* < 0.05). Overall, controlled chemical modification of egg-white proteins enables the production of sustainable, biodegradable hydrogels as promising alternatives to petroleum-based absorbent materials.

## Introduction

Abnormal eggs, including those with broken, cracked, or soft shells, arise during production due to compromised egg integrity or operational errors. These discarded eggs generated during washing and selection process are primarily applied into animal feed or liquid egg products. However, regulations restrict liquid-egg products to eggs with intact or minimally cracked shells, and the heat treatment required for sterilization further limits their potential. As egg production increase, the volume of such abnormal eggs is likely rising as well. Proteins are known for their unique functional properties, such as gelling, water-absorbing, and water-holding properties. Ovalbumin, rich in sulfhydryl groups (R-SH), undergoes aggregation and forms disulfide bonds (*S* = *S*) in response to environmental changes (Broersen et al., 2006; Li et al., 2019; Cui et al., 2025). This transition alters its structure from a fibrous linear form to a highly branched and random configuration, impacting its rheological properties. Heating under different conditions can denature ovalbumin, influencing gel turbidity and hardness through hydrophobic and electrostatic interactions (Zheng et al., 2021; Tseng et al., 2025).

Zohuriaan-Mehr et al. (2009) reported that superabsorbent polymers (SAPs) are capable of retain large amounts of water, with some capable of absorbing over 100 times their weight while maintaining their structural integrity. To enhance water absorption, hydrophilic groups can be introduced into the polymer through chemical modification (Capezza et al., 2019). Functional groups such as -COOH, -SO_3_H, -NH_2_, and -OH improve hydrophilicity by forming hydrogen bonds with water molecules (Badsha et al., 2021). The efficiency of SAPs is influenced by parameters such as polymer monomer quantity, charge density, hydrophilic group density, and cross-linking density (Sennakesavan et al., 2020). A three-dimensional network structure, achieved through cross-linking, is crucial to prevent dissolution and maintain structural integrity after water absorption. Modification of hydrophilic polymer chains increases the density of hydrophilic groups; meanwhile, environmental factors, such as pH and temperature variations also cause these groups to interact via cross-linking agents (Sennakesavan et al., 2020). Although increased cross-linking generally stabilizes the colloidal structure, it reduces hydrophilicity and create gaps between the polymers, ultimately lowering water absorption capacity. Hence, stability and water absorption of hydrogels are always inversely related in SAP production (Chavda and Patel, 2011).

Conventional absorbent materials, such as SAPs, are derived from petrochemical sources, which limits their biodegradability and reuse potential (Wilske et al., 2014). Absorbent materials play a core role in some products, like food absorbent pads to extend the shelf life of meat products. The global food absorbent pad market, valued at USD 0.86 billion in 2026 and market is projected to touch USD 1.81 billion by 2035 (Business Research Insights, 2026), is expected to generate increasing amounts of waste, primarily from polyethylene plastic materials used for moisture absorption (Khunta et al., 2025). The existing biodegradable absorbent materials showed the relatively poor swelling capacity, such as 20g/g from carboxymethyl chitosan (Shi et al., 2025) and less than 1g/g from polybutylene adipate terephthalate and thermoplastic starch films (Khunta et al., 2025). Therefore, the protein-based absorbent materials showed the high potential applied on food absorbent pads.

Egg-white powder can be converted into water-absorbent gels through alkali and heat treatments, as well as cross-linking (Rathna et al., 2024). It was reported that these biomaterial sourced gels can be degraded by fungal enzymes, demonstrating their bioavailability (Hwang and Damodaran, 1996). Acylation reagents like ethylenediaminetetraacetic dianhydride (EDTAD) and succinic anhydride (SA) are commonly used for protein modifications (Cuadri et al., 2017). Although EDTAD is effective (Senna et al., 2014), its high cost (NT$ 550/g) (Merck, 2026a), limits practical application. Conversely, SA, despite containing fewer hydrophilic groups, is more cost-effective (NT$ 2.9/g) (Merck, 2026b) and simpler to produce (Fumagalli and Updated by Staff, 2006), making it suitable for large-scale production. In term of cross-linking agents, glutaraldehyde (GA) and N, N-methylenebisacrylamide (MBA) are commonly used choices (Capezza et al., 2019). While MBA is less toxic, it is relatively expensive (NT$140/g) and classified as a hazardous substance (Acute Toxicity Category 3) (Merck, 2026cMerck, 2026c). As an alternative, glycerol (G), a widely used plasticizer, offers a cost-effective option (NT$1.7/10 mL) (Merck, 2026d). When used as a food additive for plasticization, glycerol is considered biocompatible and biodegradable. Structurally, G contains three hydroxyl groups capable of forming hydrogen bonds with polymer chains. This interaction reduces excessive cross-linking between polymer monomers while probably increasing the internal free volume, thereby enhancing the flexibility of the hydrogel (Sun et al., 2018).

Despite previous studies on egg-white or protein-based hydrogels, most have primarily focused on maximizing swelling capacity through strong chemical cross-linking, often neglecting material safety, economic feasibility, and water-holding performance under practical conditions. This study addresses these gaps by systematically comparing two acylation reagents (SA vs. EDTAD) and two cross-linking mechanisms (glycerol vs. MBA) to elucidate their distinct effects on swelling behavior, water holding capacity, reswelling performance, rheological properties, and biodegradability. Importantly, this work demonstrates that enhanced water retention and mechanical resilience can be achieved without maximizing swelling capacity, thereby challenging conventional assumptions in SAP design. In the context of agri-food waste valorization and environmental sustainability, the development of biodegradable and eco-friendly absorbent materials, i.e., SAPs, has become increasingly important. This study aimed to develop bio-absorbent hydrogels from egg-white protein using cost-effective and safe chemicals. This approach could improve the sustainability of food absorbent pads in packaging or disposable diapers by promoting recycling and reducing waste in the future.

## Materials and methods

### Materials

To ensure consistent quality of the raw material, the fresh pasteurized liquid egg white is applied for this study. It was purchased from Tai Da Eggs Technology Co., Ltd. (Taoyuan City, Taiwan) and then transported to our laboratory under 4°C. The chemical reagents used in the following experiments included: ethylenediaminetetraacetic dianhydride (EDTAD, Sigma-Aldrich, Merck KGaA Co. Inc., Darmstadt, Germany), succinic anhydride (SA, Sigma-Aldrich, Merck KGaA Co. Inc.), N,N'-methylene bisacrylamide (MBA, Sigma-Aldrich, Merck KGaA Co Inc.), ammonium persulfate (APS, Sigma-Aldrich, Merck KGaA Co Inc.), glycerol (G, Sigma-Aldrich, Merck KGaA Co Inc.), 2,4,6-trinitrobenzenesulfonic acid solution (TNBSA, 5%(w/v), Sigma-Aldrich, Merck KGaA Co Inc.), bovine serum albumin (BSA, Bio-Rad Laboratories Inc., Hercules, CA, USA), potassium bromide (KBr, Sigma-Aldrich, Merck KGaA Co Inc.), glutaraldehyde (Merck Millipore Co., Billerica, Germany), Ellman’s regent [0.04 g DNTB (5,5′-dithiobis-2-nitrobenzoic acid, Alfa Aesar, MA, USA), methanol (Honeywell Specialty Chemicals Seelze GmbH, Seelze, Germany)]. Besides, Tris-Gly buffer was composed of 0.089 M Tris (pH8.0) (Sigma-Aldrich, Merck KGaA Co Inc.), 0.09 M glycine (Sigma-Aldrich, Merck KGaA Co Inc.), 0.004 M ethylenediaminetaactic acid (EDTA, J.T. Baker; Mallinckrodt Baker, Inc., Philipsburg, NJ, USA), and 8 M urea (Sigma-Aldrich, Merck KGaA Co Inc.).

### Experimental schemes

There were two major experimental schemes in this study:

### i. Optimization of bio-absorbent hydrogel procedures with suitable concentrations of protein, succinic acid, and glycerol

Lyophilized egg white powder, which containing 0.53 g protein per g powder, was used as material in this study to ensure consistent protein concentration. Protein concentration was determined with a Bio-Rad protein assay kit (catalog #500-0006, Bio-Rad Laboratories, Inc., Hercules, CA). Protein absorbent hydrogels were prepared following the previous reported method (Cuadri et al., 2017) with slight modifications, where APS is an initiator for MBA reaction. First, the 3, 4, and 5% (w/w) protein concentrations, prepared using lyophilized egg white powder and distilled water, were selected based on the effects on water absorbency and structural integrity of the absorbent hydrogel. The treatments of different protein solution were shown in Suppl. Table 1A, and the optimal protein concentration prepared using egg white powder would be used in following experiments. Furthermore, in order to enhance manufacturing efficiency, the heating step in cross-linking reaction and the grinding step for dried hydrogels would be revised based on their effects on hydrogel swelling capacity (Suppl. Table 1B&C). In addition to simplifying the manufacturing process, this study also tended to use the safer and more cost-effective acylation (succinic anhydride, SA) or cross-linking reagent (glycerol, G) on the possibility of manufacturing protein hydrogels. The optimal SA concentration for protein hydrogel was determined as different treatments (Suppl. Table 2A). Additionally, the optimal glycerol concentration for protein hydrogel was determined as different treatments (Suppl. Table 2B). The concentration of each reagent was evaluated based on a balance of either absorbency or structural stability. The optimal concentration for each reagent was individually determined for use in subsequent experiments.

### ii. Optimal chemical modification for bio-absorbent hydrogels

As for the chemical reagent, SA and G would be tested for their applicability in absorbent-hydrogel production as alternatives to commonly used reagents, i.e., acylation reagent (EDTAD, E) and cross-linking (MBA, M). A total of 5 treatments were designed: a control group without reagent addition (X), and 4 various combinations of acylation (E and SA) and cross-linking (M and G) reagents including EDTAD plus MBA (EM), SA plus MBA (SM), SA plus G (SG), and EDTAD plus G (EG). The definitions of all experimental groups, together with their corresponding reagents and detailed dosages, were summarized in Supplementary Table 2C. The effects of these treatments on the absorbency and structural characteristics of the protein hydrogels were then evaluated.

### Analysis parameters

#### Swelling properties

The swelling capacity of the protein hydrogel was assessed using the method outlined (Hwang and Damodaran, 1996; Li et al., 2021). A tea bag containing a pre-weighed dried hydrogel was submerged in water for 24 h. After soaking, excess water was removed by allowing the tea bag to drip for 3 min. The swollen hydrogel was then reweighed, and the equilibrium swelling capacity was then calculated using the following formula:

$$\text{Equilibrium}\text{swelling}\left(g/g\right)=\left[\text{Weigh}t_{\text{swollen}\text{hydrogel}}\left(g\right)--\text{Weigh}t_{\text{dried}\text{hydrogel}}\left(g\right)\right]/\text{Weigh}t_{\text{dried}\text{hydrogel}}\left(g\right)$$

To determine the water holding capacity (%), a slightly modified procedure was referenced from the previous reported method (Pourjavadi et al., 2006). The swollen hydrogel, still within the tea bag, was transferred to a 50-mL centrifuge tube and subjected to centrifugation at 300 × *g* for 3 min using a Centrifuge 3700 (Kubota Corp., Osaka, Japan). After centrifugation, the hydrogel was weighed again, and the water holding capacity was then computed according to the following formula:

$$\begin{array}{c} \text{Water}\text{holding}\text{capacity}\left(\%\right)=\left\{\left[\text{Weigh}t_{\text{centrifuged}\text{hydrogel}}\left(g\right)--\text{Weigh}t_{\text{dried}\text{hydrogel}}\left(g\right)\right]/\text{Weigh}t_{\text{dried}\text{hydrogel}}\left(g\right)\right\}\\ /\text{equilibrium}\text{swelling}\left(g/g\right)\times 100\% \end{array}$$

#### Sulfhydryl group content

To measure sulfhydryl group content, the hydrogel was mixed with deionized water at a 1: 9 (w/w) ratio and homogenized. Subsequently, 0.05 mL of the homogeneous sample was combined with 0.7 mL of Tris-Gly buffer and 0.005 mL of Ellman’s reagent, and the mixture was heated at 40°C for 15 min. The absorbance of the solution was measured at 412 nm to determine the sulfhydryl group content (µmol eq./g protein) by conversion of coefficient of 75.53 at 412 nm with one-cm width cuvette (Feng et al., 2017).

### TNBS method and extent of modification

To evaluate the extent of chemical modification of the protein, the TNBS (2,4,6-trinitrobenzene sulfonic acid) method was employed. This method measures the amino groups that do not react with the acylation reagent, which binds to amino groups of amino acids and increases protein hydrophilicity. The procedure from a previous report (Koh et al., 2019) was adapted with minor modifications. In the analysis, hydrogel was mixed with 0.1 M NaHCO₃ at a 1:9 (w/w) ratio and homogenized for one min. The resulting protein solution was diluted to a concentration range of 0.02-0.2 mg/mL. To this solution, 0.1 mL of TNBSA (diluted to 0.01% (w/v) in 0.1 M NaHCO₃) was added, along with 0.2 mL of the sample solution or standard (BSA). After heated at 37°C for 2 h, the mixture was added 0.1 mL 10% SDS and 0.5 mL HCl (1 N) and the absorbance was measured at 335 nm to determine the content of non-bonded amino groups.

Non-bonded amino group (mmol/g) = *O*.D._335_ × 0.71 × dilution ratio × protein concentration

Here, 0.71 represents the amino group content of the standard (BSA) as reported.

Extent of modification (%) = (non-bonded amino group _X group_ − non-bonded amino group _other groups_) × 100%, where X group means the group without any reagent addition.

### Fourier transform infrared spectroscopy (FTIR)

The presence of specific chemical groups within the sample can be assessed using Fourier transform infrared spectroscopy (FTIR), which provides insights into changes in the secondary structure of proteins in hydrogel (Gao et al., 2024). For the FTIR analysis, the dried hydrogel was first ground into a fine powder. Then, 1 mg dried hydrogel powder was mixed with 0.1 g of potassium bromide (KBr). The mixture was pressed into a pellet using a pellet press (15T, Specac Co., Ltd., Orpington, UK). The FTIR spectra were recorded using a Fourier transform infrared spectrometer (Spectrum 100, Perkin Elmer Inc., Waltham, MA, USA). The spectrometer parameters were set as follows: wavelength range from 4000 to 400 cm⁻¹, resolution of 4 cm⁻¹, and 4 scans were performed for each sample.

### Rheology

The structural properties of the hydrogel were analyzed using a rheometer (HR-2, TA Instruments, New Castle, DE, USA) for a frequency sweep (Pu et al., 2022; Tseng et al., 2025). Approximately 3-5 g of the hydrogel was placed on the rheometer plate to evaluate the storage modulus (G') and loss modulus (G'') at 25°C and 5% strain, while varying the frequency from 0.1 Hz to 10 Hz. The storage modulus (G') indicates the elastic properties of the hydrogel, reflecting its solid-like behavior, whereas the loss modulus (G'') represents the viscous properties, indicative of its liquid-like behavior. The ratio of G' to G'' (tan δ) provides insight into the sample's state transition, such as a shift from a gel-like to a fluid-like state.

### Reswelling capacity

The flexibility of the hydrogel structure was assessed through cycles of swelling and dehydration to evaluate its potential for reusability. The process involved the following steps. The tea bag containing the weighted dried hydrogel was immersed in water for 24 h. After this initial swelling, the tea bag with the swollen hydrogel was transferred to a 0.15 N NaCl solution for one h to facilitate dehydration and then weighed. The dehydrated hydrogel was subsequently immersed in water for two h to allow for re-swelling. The cycle of swelling and dehydration was repeated, and the reswelling capacity was calculated based on the number of dehydration cycles.

### Microstructure (scanning electron microscope, SEM)

In the scanning electron microscope, the reflected electron beam hits the surface of the sample, and the resulting signals are detected by the detector and converted into an image. This technique is primarily used to examine the surface morphology, particle arrangement, and the size and shape of various samples. For the analysis, swollen hydrogel samples were cut into 3 × 1 × 1 mm pieces and immersed in 2.5% glutaraldehyde buffer overnight to fix the structure. Following fixation, the samples were washed 3 times with 0.1 M phosphate-buffered saline (PBS), with each wash lasting 10 min. Subsequently, the samples were dehydrated through a graded series of ethanol solutions (50%, 60%, 70%, 85%, 90%, 95%, 100%, 100%, and 100%), with each ethanol gradient applied for 10 min. The dehydrated samples were then subjected to critical point drying using a critical point dryer (Samdri-PVT-3B, Tousimis Co., Inc., Rockville, MD, USA) for approximately two h. Both the processed swollen hydrogel samples and dried hydrogel samples (3 × 1 × 1 mm) were placed on a gold-plated sample holder and coated with a thin layer of gold using a sputter coater (Iron Sputter, SPI, West Chester, PA, USA). The final samples were examined using an SEM (JSM-6510LV, JEOL Ltd., Akishima, Tokyo, Japan) at magnifications of 500X, 1500X, and 20,000X to assess their surface characteristics.

### Biodegradation

An accelerated biodegradability test was performed to compare the environmental friendliness of petroleum-based gels (water beads) and protein-based hydrogels within a short observation period. The assay employed a nutrient- and microbe-rich culture soil as degradation media (Sarmah and Karak, 2020). Specifically, 18 g of culture soil (Sinon Corporation, Taichung, Taiwan) was added into a 50 mL centrifuge tube. An empty, dry tea bag was first weighed, then filled with the water beads or hydrogel sample and reweighed. Subsequently, 20 g of deionized water was added to the tube. The sample-containing tubes were incubated at 40 °C for various durations (7, 14, and 21 days) to assess the extent of degradation over time. After incubation, the tea bags were removed, gently rinsed with deionized water to eliminate excess soil, and soaked in deionized water for one h to further remove residual particulates. The tea bags were then dried in a 40 °C oven for 24 h. Once completely dried, the tea bags were reweighed. The percentage of biodegradation was calculated by the weight change.

Biodegradation (%) = [Weight degraded hydrogel with tea bag (g) – Weight fresh dried hydrogel with tea bag (g)]/Weight fresh dried hydrogel with tea bag (g) × 100%

### Statistical analysis

Protein hydrogels were prepared in three independent batches, with each batch subjected to a minimum of three replicates. All experimental results were presented as mean ± SEM (standard error of the mean). Differences on swelling capacity of hydrogel production subjected to heating or grinding steps were analyzed using Student’s t-test (*p* < 0.05). Other experimental data were obtained from studies designed as a completely randomized design (CRD). When a significant difference (*p* < 0.05) among groups was analyzed by using ANOVA, the least significant difference (LSD) analysis was used for post-hoc analysis to identify specific differences between treatment groups (*p* < 0.05). All statistical data analyses were performed using Excel or SAS software (SAS Institute Inc., Cary, NC, USA).

## Results and discussion

### Optimization of manufacturing process and alternative chemical-reagent concentrations for egg-white absorbent hydrogels

#### Optimization of manufacturing process

##### Determination of suitable protein concentration prepared with egg white

**Fig. 1:**
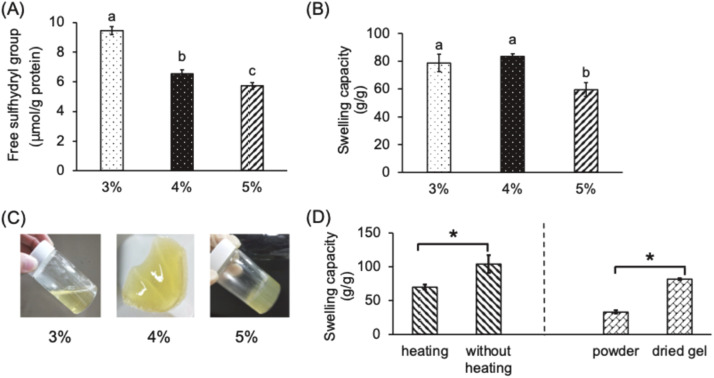
Effects of various protein concentrations prepared with egg white on (A) free sulfhydryl group content, (B) swelling capacities, and (C) appearances of egg-white based hydrogels as well as effects of heating process during the acylation process and grinding of final hydrogel on (D) swelling capacities of egg-white based hydrogels. 1. The data are given as mean ± SEM (*n* = 3). Data bars in each test parameter without a common letter are significantly different (*p* < 0.05). 2. *: *p* < 0.05 heating (60°C heating for 30 min) vs. without heating and powder vs dried gel, respectively.

According to the manufacturing protocol for egg-white hydrogels (Rathna et al., 2024), 1% protein (w/w) solution was initially prepared with lyophilized egg-white powder. The protein solution was adjusted to pH 12 using 10 N NaOH, followed by heating at 60 °C for 30 min. Subsequently, the acylation reagent (EDTAD) was added to induce chemical modification to increase hydrophilicity. The resulting solution was then lyophilized to obtain the “modified egg-white powder”. This modified powder was redissolved to a concentration of 15% (w/w) and subjected to thermal treatment at 80 °C for 60 min to induce gelation. The formed gel was dehydrated at 50 °C for 24 h, and then ground into powder for subsequent experiments. The purpose of second-round of lyophilization is originally to increase protein concentration for gelation and reduced storage stress; however, it could be omitted from our manufacturing process due to the poor solubility observed in 15% “modified egg-white powders” (Suppl. Fig. 1). As we know, during freeze-drying, egg-white powder is subjected to various stresses, including fluctuations in solute concentrations, ice crystal formation, and pH shifts, which may induce varying degrees of protein denaturation, thereby reducing solubility. In the absent of this step, a second redissolution is unnecessary, allowing the determination of protein concentration at the initial stage. Sequentially, 3, 4, and 5% protein (w/w) concentrations for redissolving protein powder were set in this study. After gel formation from the resulting protein solutions, their sulfhydryl (R-SH, thiol group) content and water absorption were assayed. As shown in Fig. 1A, a significant reduction (*p* < 0.05) in sulfhydryl group content from 9.45 to 5.72 μmol/g protein was assayed as the protein concentration increased from 3 to 5%. Additionally, gels prepared with 3 and 4% protein (approximately 78.7 and 83.4 g water/g gel, respectively) exhibited superior (*p* < 0.05) water absorption compared to the 5% gel (64.8 g water/g gel), with no (*p* > 0.05) difference observed between 3 and 4% protein formed gels (Fig. 1B). Meanwhile higher protein concentrations resulted in stronger and more stable colloidal gel structure (Fig. 1C). Changes in environmental conditions, such as pH and temperature, can induce protein denaturation. This process can lead to alterations in the protein’s quaternary and tertiary structures, potentially reverting them to secondary or primary structures. Consequently, functional groups within the protein become exposed, and some covalent bonds may break, generating free groups. Specifically, in ovalbumin, disulfide bonds may break to reverse to sulfhydryl groups, which can subsequently re-form disulfide bonds with sulfhydryl groups from other protein chains to structure the 3D network hydrogel (Zhao et al., 2016). Thus, the content of sulfhydryl groups serves as an indicator of sulfhydryl-disulfide exchange, providing insight into hydrogel formation. Increased protein concentration brings molecules closer, promoting disulfide bond formation and reducing sulfhydryl groups, thereby enhancing colloidal strength (Chen et al., 2021). This increased strength correlates with greater structural rigidity, supporting the structural integrity of hydrogels prepared with different protein concentrations. Water absorption is influenced not only by protein concentration but also by gel structure and hydrophilicity. A higher protein concentration in a 5% colloid results in denser protein interactions and fewer voids for water entering, reducing water absorption. Conversely, although the water absorption of the 4% colloid is similar to the 3% colloid, the higher protein content in the 4% gel involves more protein in the acylation reaction, increasing hydrophilic groups and enhancing water absorption compared to the 3% hydrogel. As illustrated in Fig. 1A-C, the 4% protein solution prepared with lyophilized egg-white powder formed an absorbent hydrogel with higher swelling capacity and adequate structure strength. The reduced sulfhydryl group (R-SH) content indicates the formation of more disulfide bonds, contributing to the gel network stability.

##### Impacts of omitting pre-heating and grinding

The protein solution, pre-treated by pH adjustment to 12, was directly utilized to use for protein denaturation in the continuous steps without lyophilization again. That heating step (60 °C for 30 min) is necessary or not was evaluated to improve procedural efficiency. The swelling capacity of the hydrogel prepared with the heating step was 74.5 g water/g, which was significantly lower than that of the hydrogel prepared without heating (91.1 g water/g, *p* < 0.05) (Fig. 1D). This result indicates that the extent of protein denaturation induced by alkaline treatment alone was sufficient to form a stable hydrogel network, thereby eliminating the need for the pre-heating step at 60 °C for 30 min (Rathna et al., 2024). Ovalbumin, the primary protein in egg whites, contains disulfide bonds and 4 free sulfhydryl groups within its hydrophobic core (Broersen et al., 2006). In the highly alkaline environment (pH12) used initially, denaturation exposes sulfhydryl groups and reduces some disulfide bonds to sulfhydryl groups (Zhao et al., 2016). An increase in pH enhances electrostatic repulsion between protein molecules, thereby promoting a greater extent of denaturation. This includes conformational changes, increased exposure of free sulfhydryl groups, improved solubility, and reduced surface hydrophobicity (Zhang et al., 2023). Hence, it is speculated that the highly alkaline environment could induce substantial egg-white protein unfolding, exposing numerous free amino groups that subsequently react with the acylation reagent, thereby enhancing the water absorbency of hydrogel. Therefore, the revised procedures not only simplified the experimental workflow by eliminating a thermal treatment step but also resulted in a hydrogel with improved functionality. Besides, particle size is related to the initial absorption rate, instead of the total absorption volume (Abdallah, 2019). As shown in Fig. 1D, the water absorbency of the dried hydrogel product was significantly decreased after grinding (powder form) (*p* < 0.05). This reduction might be attributed to the fact that, at the same level of chemical modification, i.e., consistent numbers of hydrophilic groups, the vital factor influencing water absorbency is the available physical space within the hydrogel matrix for water retention. Therefore, forming the hydrogel into plate molds may eliminate the need for a grinding step, thereby simplifying the manufacturing process and increasing batch consistency.

Based on our current results, the protein-absorbent hydrogel prepared from egg white can be produced as following description. Egg white is first lyophilized to obtain a powder, which is then used to prepare a 4% protein solution. The pH of this solution is adjusted to 12 using NaOH for 30 min. To ensure proper acylation, the pH value is further adjusted according to the specific acylation reagent, such as pH12 for EDTAD or pH8 for SA, based on the requirements of the following experiment (Hwang and Damodaran, 1996; Cuadri et al., 2017). The solution is then reacted with the selected acylation reagent for 2.5 h. After foam is removed by filtration, a cross-linking reagent, e.g., MBA or G, is added to the acylated protein solution and stirred for 10 min. The resulting mixture is then casted into a plate mold and then heated in a water bath at 80°C for 40 min to induce gelation. Finally, the hydrogel is then dried at 50°C for 24 h to obtain the final product.

##### Optimal concentrations of either alternative acylation or cross-linking reagents for egg-white absorbent hydrogels

**Fig. 2:**
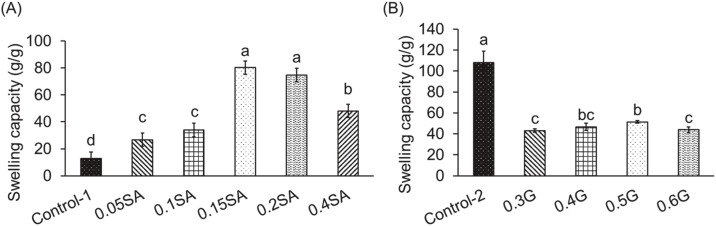
Effects various concentrations (w/w) of (A) succinic anhydride (SA) and (B) glycerol (G) on swelling capacities of egg-white based hydrogels. 1. The data are given as mean ± SEM (*n* = 3). Data bars in each test parameter without a common letter are significantly different (*p* < 0.05). 2. (A): SA (succinic anhydride) addition as an acylation reagent and MBA (N,N’-methylene bisacrylamide)+APS (ammonium persulfate) addition as a cross-linking reagent. Control-1: only MBA+APS addition. 3. (B): EDTAD (ethylenediaminetetraacetic dianhydride) addition as an acylation reagent and G (glycerol) addition as a cross-linking reagent. Control-2: only EDTAD addition.

Followed by previous methods for protein hydrogel process, the control groups utilized acylation and cross-linking reagents consisting of 0.2 g EDTAD/g protein (Hwang and Damodaran, 1996) and 0.015 M MBA (0.13 g MBA/g protein) with 0.062 g APS/g protein as the initiator (Feng et al., 2017), respectively. The critical parameter for dosage selection was water absorption capacity, the key function of protein hydrogels. Fig. 2A shows that with 0.13 g MBA + 0.062 g APS/g protein as the cross-linking reagent, the hydrogel with 0.15 and 0.2 g SA/g protein exhibited the highest water absorption capacity of 80.19 and 74.68 g/g, followed by 0.4 g SA/g, 0.1 g SA/g, 0.05 g SA/g, and Control-1 group (*p* < 0.05). Regarding chemical dose selection, both SA and MBA react with amine groups on proteins to form bonds (Capezza et al., 2019). This suggests a competitive interaction between them. Adding an excess of SA beyond the amount of available amine groups in the protein may not positively significantly affect the colloidal structure or water absorption. However, an excessive amount of SA can lead to the formation of intermolecular hydrogen bonds between hydrophilic groups, potentially altering the gel structure and reducing its water absorption capacity (Kaur et al., 2023). Due to the same result by adding 0.15 or 0.2 g SA/g protein, 0.15 g SA/g protein was chosen to lower cost for use comparison in subsequent experiments. Additionally, tests with 0.2 g EDTAD/g protein as the acylation reagent revealed that the use of glycerol (G) as a cross-linking reagent reduced (*p* < 0.05) the water absorption capacity of the protein hydrogel (Fig. 2B). However, protein hydrogels without G addition as a cross-linking reagent exhibited hydrogel structure formed hardly compared to Control-2 (without crossing-linking reagent). Among the glycerol formulations, the hydrogel prepared with 0.5 g glycerol/g protein had the highest (*p* < 0.05) water absorption (51.43 g/g) compared to other glycerol-containing groups, although this was still lower than the Control-2 group (107.99 g/g). The difference was statistically significant compared to the other glycerol-added treatments, except for the 0.4 g glycerol/g protein group (*p* < 0.05). Chen et al. (2022) reported that insufficient or lower doses of cross-linking reagents may hinder the formation of a stable colloidal structure necessary for water retention. Conversely, excessive amounts can overly tighten the polymer network, reducing the available space within the colloid matrix and thereby decreasing water absorption. Based on these findings, 0.5 g glycerol/g protein could be selected as the optimal level of the cross-linking reagent for building a robust colloidal structure in further experiments. In subsequent experiments, the experimental hydrogels were divided into 5 groups: X group [no reagents], EM group [0.2 g EDTAD/g protein + (0.13 g MBA+0.062 g APS)/g protein], SM group [0.15 g SA/g protein + (0.13 g MBA+0.062 g APS)/g protein], SG group [0.15 g SA/g protein + 0.5 g G/g protein], and EG group [0.2 g EDTAD/g protein + 0.5 g G/g protein]. The EM formulation was served as the positive control to assess the impact of these chemical treatments on the egg-white based protein hydrogel.

##### Comparison of swelling properties of hydrogels under different acylation and cross-linking reagent combinations

Since the primary function of absorbent hydrogels is water absorption, subsequent experiments focused on evaluating their swelling properties. Furthermore, the degree of chemical modification and FTIR results were analyzed to elucidate the influence of various combinations with both acylation and cross-linking agents on hydrogel’s water absorption.

##### Water absorbency of hydrogels under different acylation and cross-linking reagent combination

**Fig. 3:**
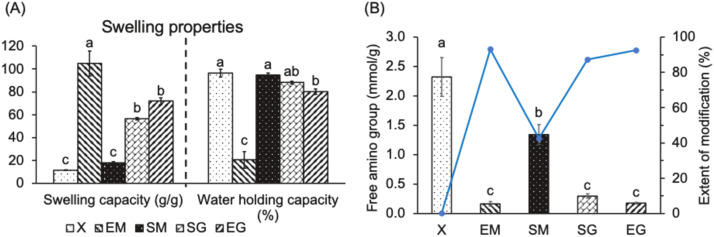
Effects of different acylation and cross-linking-reagent combinations on (A) swelling properties, as well as (B) the extent of free amino groups and modification of lysyl residues of egg-white based hydrogels. 1. The data are given as mean ± SEM (*n* = 3). Data bars in each test parameter without a common letter are significantly different (*p* < 0.05). 2. X: without any reagent; EM: EDTAD (ethylenediaminetetraacetic dianhydride) + MBA (N,N’-methylene bisacrylamide)/APS (ammonium persulfate); SM: SA (succinic anhydride) +MBA/APS; SG: SA+*G* (glycerol); EG: EDTAD+*G*.

Fig. 3A shows that the EM group exhibits the highest swelling capacity (104.7 g/g), significantly greater than that of the other groups (*p* < 0.05). The SG and EG groups had secondly higher (*p* < 0.05) swelling capacities of 56.6 and 71.9 g/g, respectively, with no significant difference between them (*p* > 0.05). Conversely, the SM and X groups demonstrated the lowest water absorption, with values of 17.9 and 11.6 g/g, respectively (*p* < 0.05). Fig. 3A also reveals that the X and SM groups had the highest water-holding capacities (96.5% and 94.9%, respectively), followed by the SG and EG groups (88.2% and 80.2%, respectively), with the EM group showing the lowest capacity (20.4%) (*p* < 0.05). The minimal water absorption in the X and SM groups results in higher water retention after centrifugation. In contrast, it is speculated that glycerol in the SG and EG groups enhances colloidal flexibility and water-holding capacity. The significantly lower water-holding capacity of the EM group is likely due to excessive water absorption, which compromises the colloidal structure's stability and water-retention ability.

##### Chemical modification of hydrogels under different acylation and cross-linking reagent combinations

The degree of acylation of the protein in egg white can be inferred by measuring the number of amine groups. Fig. 3B shows that the amine content in the protein hydrogels of the EM, SG, and EG groups (0.16, 0.30, and 0.18 mmol/g protein, respectively) was significantly lower than that in the X and SM groups (2.33 and 1.34 mmol/g protein, respectively) (*p* < 0.05). The X group, with no chemical addition, served as the benchmark for total amine groups. Calculations indicated that the degree of chemical modification in the EM, SG, and EG groups is approximately 90%, suggesting these groups have a higher number of hydrophilic groups on their polymer chains, which enhances their swelling capability (water absorption). Based on the chemical structures of the acylation reagents, EDTAD and SA, used in this study, both reagents interact with the amino groups on proteins in a highly alkaline environment, thereby increasing the number of hydrophilic groups on the polymer chains (Hwang and Damodaran, 1996; Pavliakova et al., 1999). Since EDTAD and SA are acylation reagents, the water absorption of the colloid is primarily influenced by the content of hydrophilic groups (COO-) introduced by these reagents. Given that EDTAD contains more hydrophilic groups than SA (Capezza et al., 2019), if both reagents have equivalent modification effects on proteins, the group containing EDTAD is expected to exhibit higher water absorption. Moreover, an excessive degree of cross linking in the gel can reduce its expansion capacity upon water absorption. However, an insufficient cross linking may result in an incomplete gel structure, impairing its water-absorbing functionality which exhibits a low water holding capacity. Therefore, achieving an optimal balance between chemical modification (acylation) and cross linking is crucial. Acylation agents (EDTAD or SA) and cross-linking agents (MBA) form covalent bonds with the amino groups on proteins (Capezza et al., 2019; Wang et al., 2024). Based on the observed differences in absorbency and water retention, a plausible explanation is that competitive interactions between EDTAD or SA and MBA may occur during the modification process. Fig. 3B reveals that the residual free amino group in both EG and SG groups is below 10%, confirming the high affinity of EDTAD (E) and SA (S) for protein amino sites, regardless of glycerol addition. The cross-linking agent (G), which interacts with proteins via hydrogen bonds (Sun et al., 2018), its interaction with the acylation agent is minimal. The SG and EG groups, which incorporate G, showed higher water absorption, increased cross linking, and improved water-holding capacity (Fig. 3A). The EM group exhibits a free amino group comparable to that of the EG group (< 10%), suggesting that EDTAD effectively occupies amino sites even in the presence of MBA (M) (Fig. 3B). However, this preferential sequestration by EDTAD may hinder the ability of MBA to establish a coherent network, resulting in a hydrogel characterized by superior swelling capacity but attenuated water retention. In this context, it is hypothesized that EDTAD exhibit a stronger or more dominant interaction with protein amino groups in the EM group than MBA, which may contribute to higher water absorption but lower water retention, with a larger fraction of the absorbed water existing as free water. Conversely, the different free amino group content between SM and SG groups indicates that MBA competitively interferes with the SA-amino interaction, causing the displacement of SA and the subsequent liberation of amino groups. These results suggested that the binding affinity of MBA for amino groups is superior to that of SA. The displacement by MBA facilitates the formation of a more densely cross-linked architecture, which accounts for the observed enhancement in water retention and the corresponding reduction in water absorption. However, this interpretation is inferred from indirect observations and would require further characterization to be directly confirmed.

##### Secondary structure of hydrogels under different acylation and cross-linking reagent combinations

**Fig. 4:**
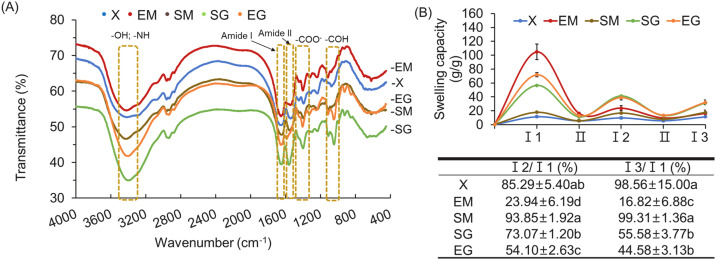
Effects of different acylation and cross-linking-reagent combinations on (A) FTIR spectra and (B) reswelling capacities of egg-white based hydrogels. 1. The data are given as mean ± SEM (*n* = 3). Mean in each test parameter without a common letter are significantly different (*p* < 0.05). 2. X: without any reagent; EM: EDTAD (ethylenediaminetetraacetic dianhydride) + MBA (N,N’-methylene bisacrylamide)/APS (ammonium persulfate); SM: SA (succinic anhydride) +MBA/APS; SG: SA+*G* (glycerol); EG: EDTAD+*G*. 3. 3400 cm^-1^ (3100-3500 cm^-1^): -OH and -NH, 2930 cm^-1^: -CH, 1640 cm^-1^: -*C* = *O* (aldehydes)and -CN (amide I), 1550 cm^-1^: -NH and -CN (amide II), 1405 cm^-1^: -COO^-^ (carboxyl group), 1045 cm^-1^: -COH (alcohols). 4. Ⅰ1, Ⅰ2, Ⅰ3: swollen in water. Ⅱ: swollen in 0.15 N NaCl.

Based on the infrared spectroscopy absorption Table (Socrates, 2004; Mir et al., 2023), absorption peaks in infrared spectra correspond to specific stretching or bending vibrations of chemical functional groups. A red shift in the absorption peak indicates a longer wavelength and lower frequency, corresponding to reduced energy and weaker bonding between proteins (Rozenberg et al., 2005). Conversely, a blue shift reflects a shorter wavelength and higher frequency, suggesting increased energy and stronger bonding. Fig. 4A illustrates the chemical functional group composition of protein hydrogels produced with various chemical combinations, as analyzed by FTIR spectroscopy. The prominent absorption peaks include: 3400 cm⁻¹ (3100–3500 cm⁻¹) for -OH and -NH groups; 2930 cm⁻¹ for -CH groups; 1640 cm⁻¹ (amide I) for -*C* = *O* and -CN groups; 1550 cm⁻¹ (amide II) for -NH and -CN groups; 1405 cm⁻¹ for -COO groups; and 1045 cm⁻¹ for -COH groups (Fig. 4A). At ∼ 3400 cm⁻¹, the absorption peak positions for -OH and -NH groups were as followings: 3421 cm⁻¹ (X), 3400 cm⁻¹ (SG), 3402 cm⁻¹ (EG), 3434 cm⁻¹ (SM), and 3422 cm⁻¹ (EM). Compared to the X group, SG and EG groups showed a red shift, indicating weaker hydrogen bonding, whereas SM and EM groups exhibited a blue shift, implying stronger bonding. This phenomenon is typically associated with increased hydrophobic interactions (Pu et al., 2022). The absorption peak at 1405 cm^-1^ corresponds to the -COO⁻ group, a key hydrophilic moiety, exhibited a blue shift in SG, EG, SM, and EM groups compared to the X group, suggests enhanced bonding interactions between the protein and this hydrophilic group. Additionally, changes in the amide I and II peaks reflect variations in peptide bond content. Specifically, a decrease in amide I coupled with an increase in amide II suggests an increase in peptide-bond formation (Cuadri et al., 2017). The protein's secondary structure can be assessed through the amide I (1640 cm^-1^) and amide II (1550 cm^-1^) peaks (Ye et al., 2014). Amide I primarily arise from -*C* = *O* stretching vibrations (∼80%), with -CN stretching contributing less, while amide II results from -N-H bonding (∼60%) and -CN stretching (∼40%) (Jackson and Mantsch, 1995). Thus, shifts and intensity changes in these bands can be used to infer variations in peptide bond content (Cuadri et al., 2017). The observed amide I peak positions were 1653 cm⁻¹ (X), 1649 cm⁻¹ (SG), 1652 cm⁻¹ (EG), 1642 cm⁻¹ (SM), and 1654 cm⁻¹ (EM). For amide II, they were 1543 cm⁻¹ (X), 1563 cm⁻¹ (SG), 1587 cm⁻¹ (EG), 1551 cm⁻¹ (SM), and 1543 cm⁻¹ (EM). Notably, the EM group exhibited a blue shift in amide I with no change in amide II, suggesting a possible reduction in peptide bonds. In contrast, the other groups showed shifts indicative of increased peptide bonds formation compared to the X group. Besides, pronounced absorption peaks at 1045 cm⁻¹ (-COH) was observed only in both SG (1046 cm⁻¹) and EG (1047 cm⁻¹) groups, which contains glycerol. The prominent peaks in SG and EG suggest that successful binding contributes to structure stabilization. Based on these observations, the structural characteristics of the different hydrogel samples can be inferred. EM group has fewer peptide bonds but stronger hydrogen bonds between proteins and more hydrophilic groups, corresponding to a less stable colloidal structure (lower water holding capacity) but strong water absorption. SM group exhibits higher peptide bond contents, stronger hydrogen bonds between proteins, and fewer hydrophilic groups which results in a strong colloidal structure with limited water absorption. SG group shows more peptide bonds, weaker protein-protein hydrogen bonding between proteins, and fewer hydrophilic groups where hydrogen bonding, and fewer hydrophilic group, demonstrating a more stable structure but moderate water absorption. EG group contains more peptide bonds, fewer hydrogen bonds between proteins, and higher hydrophilicity due to glycerol, contributing to a strong colloidal structure and strong water absorption.

##### Comparison of structure property of hydrogels under different acylation and cross-linking reagent combinations

Since the stability of the hydrogel network inversely correlates with water absorption, evaluating an absorbent hydrogel requires assessing both its hydrophilic group content and network structure. This section evaluated hydrogel-network strength through reswelling capacity, rheological measurements, and microstructural analysis.

##### Reswelling capacity of hydrogels under different acylation and cross-linking reagent combinations

Fig. 4B illustrates hydrogel-network stability across different groups. During stage II, osmotic pressure differences between saline and water caused protein hydrogels to dehydrate. Upon rehydration, water absorption levels in stages I2 and I3 varied, reflecting differences in hydrophilicity. The top image of Fig. 4B shows that, despite a gradual decrease in reswelling capacity after multiple absorption-dehydration cycles, the SG and EG groups retained higher reswelling capacities compared to other groups. The EM group showed the greatest water initial absorption but exhibited a rapid decrease in reswelling capacity following dehydration. However, both X and SM groups absorbed less water initially and still kept lower swelling capacities compared to the other groups after dehydration. Based on the bottom of Fig. 4B, the reswelling capacities (%) of each group, reflect changes in water absorption ratios over different periods. The absorption ratios (I2/I1) for the X and SM groups were 85.29 and 92.85%, respectively, increasing to 98.56% and 99.31% in I3/I1. These groups demonstrated significantly higher resorption rates compared to others (*p* < 0.05), primarily due to their low initial water absorption. Their stable and rigid colloidal structures during absorption-dehydration cycles contributed to their improved and consistently high resorption rates. The absorption ratios (I2/I1) for the SG and EG groups were 73.07 and 54.10%, respectively, significantly lower than the SM group but higher than the EM group (*p* < 0.05). For ratios in I3/I1, SG and EG decreased to 55.58 and 44.58%, respectively, also significantly lower than the SM and X groups but still higher than the EM group (*p* < 0.05). As indicated by FTIR (Fig. 4A), it is speculated that the structural stability of SG and EG groups relies on the formation of peptide bonds and hydrogen bonds between hydroxyl groups (-OH) from glycerol rather than hydrogen bonds between proteins. The total bond energy is weaker than that found in SM and X groups (peptide bonds and hydrogen bonds between proteins). It shows that the structure of SG and EG have relative weak hydrophobic interactions between protein and much carboxyl groups (COO-) to absorb water, so their colloidal structures are less rigid but more elastic. In the EM group, the absorption ratios of I2/I and I3/I2 were the lowest among all groups (*p* < 0.05). Despite its high initial water absorption, the hydrogel-network structure of the EM group became compromised as excessive water is absorbed, leading to inadequate basic water retention and a decrease in subsequent reabsorption capacity. FTIR suggests that the EM group primarily relies on hydrogen bonds between proteins and has fewer peptide bonds, making its hydrogel-network structure less stable and prone to disintegration. Although it remains highly hydrophilic, its inability to retain water negatively impacts measurements, rendering samples with low water-retention capacity unsuitable for commercial applications. Based on the results, the SG group maintained a reswelling capacity above 50% even after two dehydration cycles, indicating that although dehydration can damage the protein hydrogel structure, it still retained a baseline level of water-absorption and retention capability with a relatively stable structure.

##### Rheological properties of hydrogels under different acylation and cross-linking reagent combinations

**Fig. 5:**
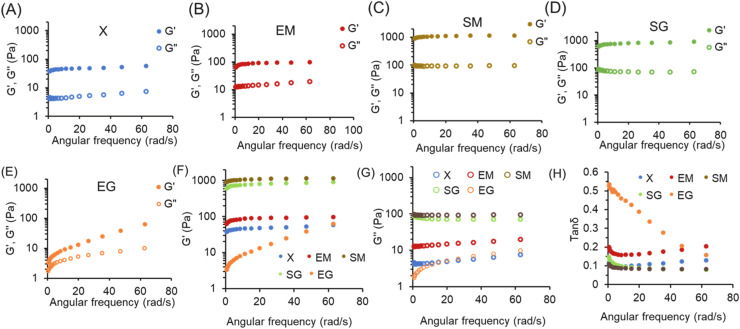
The rheological properties of egg-white based hydrogels prepared with different chemical reagent combinations: (A)∼(E): G’ and G’’ value of egg-white based hydrogels prepared with different acylation and cross-linking reagent combinations, and (F)∼(H): G’, G’’ value, and tanδ of different egg-white based hydrogels. 1. X: without any reagent; EM: EDTAD (ethylenediaminetetraacetic dianhydride) + MBA (N,N’-methylene bisacrylamide)/APS (ammonium persulfate); SM: SA (succinic anhydride) +MBA/APS; SG: SA+*G* (glycerol); EG: EDTAD+*G*.

Fig. 5A–E illustrate that protein gels treated with each chemical reagent formed solid gels after heat treatment. Across all groups, the storage modulus (G') was consistently higher than the loss modulus (G''), confirming gel formation. Although the EG group showed G' values (3.28 Pa) slightly higher than G'' (1.73 Pa), the difference was not discussed in comparison with other groups, and the values were too low to clearly determine the material’s state. In contrast, the G' values of the other 4 groups were substantially higher than their own G'' (e.g., SG: G': 601.25 Pa, G'': 88.32 Pa; SM: G': 897.54 Pa, G'': 98.44 Pa) and changed relatively slowly with frequency variation. This rheological stability suggests strong internal bonding within their colloidal structures, maintaining a stable gel state. The EG group displayed significant variations in G' and G'' with frequency changes, suggesting that bond stabilization was incomplete after heat treatment. The more pronounced increase in G' compared to G'' indicates ongoing bond formation. As a result, the EG group remained in an unstable gel state following a heat treatment. Fig. 5F and G compared the initial G' and G'' values for each group to assess the gel state after heat treatment. The SM and SG groups exhibited significantly higher G' values (897.54 Pa and 601.25 Pa, respectively) compared to the EM and X groups (63.15 Pa and 37.55 Pa, respectively). Similarly, the higher G'' values for SM and SG (98.44 Pa and 88.32 Pa, respectively) were also observed, though the difference was less pronounced. In contrast, G'' values for EM and X were much lower (12.60 Pa and 4.29 Pa, respectively). The tan δ ratio, which indicates the viscoelastic properties, shows that lower tan δ values reflect more solid-like behavior, while a tan δ value exceeding 1 suggests a transition from a solid to a fluid state. As shown in Fig. 5H, the tan δ values for the SM, SG, and X groups were relatively low (0.11, 0.15, and 0.11, respectively), indicating a solid-like behavior. The EM group had a tan δ value of 0.20, while the EG group exhibited a significantly higher initial tan δ value of 0.53, which decreased with increasing frequency. Based on these results, the SM and SG groups exhibit similar elastic solid gel characteristics. The EM group behaves as viscoelastic solid gel with higher viscosity, the X group shows greater elasticity in its viscoelastic solid gel, and the EG group behaves highly viscous colloid.

According to FTIR spectra, the SM group exhibits stronger peptide and hydrogen bonds between proteins, while the SG group has more peptide and hydrogen bonds formed between hydroxyl group (-OH), with fewer hydrophilic groups (carboxyl group, -COO-) (Fig. 4A). This facilitates the formation of an elastic gel state upon heating. The SG group's higher water absorption compared to the SM group (Fig. 3A) allows water to enter and stabilize its colloidal structure, promoting network formation. The EM group features stronger hydrogen bonds between proteins (Fig. 4A), resulting in a harder colloidal structure and the formation of a viscoelastic solid gel. Conversely, the EG group, with more peptide bonds and hydrogen bonds formed between alcohol groups (Fig. 4A), also both groups contain more hydrophilic groups (carboxyl groups, -COO-). Despite this, excessive hydrophilicity in the EM group (Fig. 3A) leads to structural breakdown after water absorption. The presence of glycerol in the EG and SG groups, a plasticizing cross-linking agent, enables significant water reabsorption while maintaining structural flexibility. In addition, the EG group showed a fluid-like colloid shape before drying, indicating that bonds occur progressively during drying process to stabilize the gel network, with full structural development achieved only after drying. Therefore, complete drying plays a critical role in the gel's water absorption properties.

##### Microstructure of dried and swollen hydrogels under different acylation and cross-linking reagent combinations

**Fig. 6:**
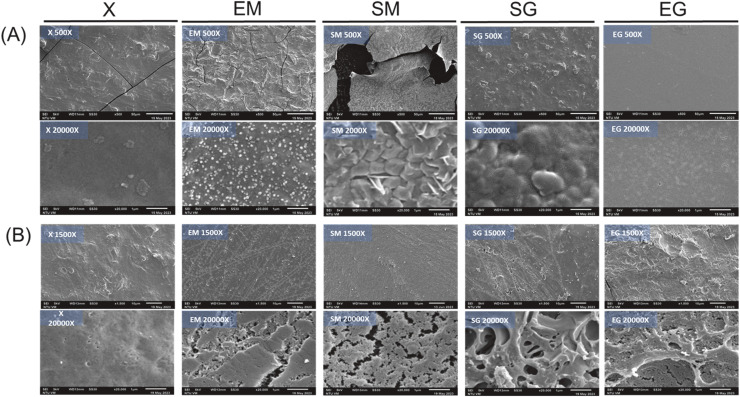
The SEM images of (A) dried and (B) swollen egg-white based hydrogels prepared different chemical reagent combinations. 1. X: without any reagent; EM: EDTAD (ethylenediaminetetraacetic dianhydride) + MBA (N,N’-methylene bisacrylamide)/APS (ammonium persulfate); SM: SA (succinic anhydride) +MBA/APS; SG: SA+*G* (glycerol); EG: EDTAD+*G*.

Electron microscopy provides qualitative information on surface morphology and structural features of protein hydrogels before and after water absorption, offering insight into how different chemical treatments influence structural integrity. However, it should be noted that SEM mainly reflects localized surface morphology and does not directly represent the three-dimensional pore structure or bulk porosity of the gels. Fig. 6A showed the microstructures of dried hydrogels. The X group, which received no chemical treatment, exhibited a relatively smooth and crack-free surface at both macroscopic (500X) and microscopic (20,000X) magnifications. In contrast, the SM and EM groups displayed uneven surfaces with numerous cracks at the macroscopic scale, suggesting increased susceptibility to drying-induced structural damage following chemical modification. At higher magnification, these cracks likely reflect fragmentation or shrinkage effects occurring during dehydration rather than intrinsic porosity differences. Egg protein gels are primarily stabilized by disulfide bonds, while MBA further introduces covalent cross-links through reactions with amine groups. The combined presence of these covalent interactions may increase network rigidity, rendering the SM and EM gels less tolerant to shrinkage stress during drying and thus more prone to cracking. Although MBA has been reported as an effective cross-linking agent in soy protein-based hydrogels (Prusty et al., 2019), soy protein networks are mainly stabilized by weaker non-covalent interactions. In contrast, protein systems dominated by covalent bonding may respond differently to additional cross-linking, resulting in excessive rigidity. Conversely, the SG and EG groups exhibited relatively smooth and continuous surfaces after drying. The presence of glycerol likely promotes hydrogen bonding with protein chains, reducing intermolecular bonding energy and increasing structural flexibility. This plasticizing effect may help preserve structural integrity during dehydration and mitigate crack formation. Fig. 6B presents the microstructures of swollen hydrogels. The X group maintained a smooth and intact surface at both magnifications, indicating a mechanically stable structure with limited swelling, consistent with its low water uptake measured experimentally. The absence of visible cracks suggests minimal structural deformation upon hydration rather than enhanced water-holding capacity. In contrast, the EM and SM groups showed pronounced surface cracking after swelling, indicating that water absorption induced structural disruption. Although the EM group exhibited high initial water uptake, the damaged structure likely compromises its ability to retain water over time. Similarly, the rigid network of the SM group limits both swelling and effective water retention. The SG and EG groups displayed more open and interconnected surface features after swelling, with a sponge-like morphology observed at higher magnification. While these features should not be interpreted as direct measures of pore size or porosity, they qualitatively reflect a more deformable and hydrated network structure. Combined with water-holding capacity measurements, these observations suggest that the SG and EG gels possess a balanced network architecture that accommodates water while maintaining structural stability. Such networks may enhance water retention through capillary effects and elastic resistance to deformation (Pu et al., 2022).

##### Difference of biodegradation between protein-based hydrogel and petroleum-based gel

**Fig. 7:**
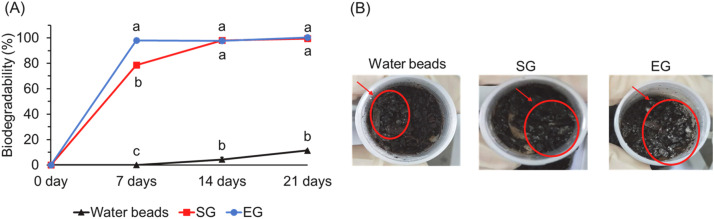
(A) The biodegradability in soil with different hydrogels. (B) The bacterial growth in soil with water beads, and SG and EG hydrogels in 7 days. 1. The data are given as mean ± SEM (*n* = 3). Data bars in each test parameter without a common letter are significantly different (*p* < 0.05). 2. SG: SA (succinic anhydride) +*G* (gl;ycerol). EG: EDTAD(ethylenediaminetetraacetic dianhydride) + *G*.

To simulate environmental degradation conditions (ecofriendly), culture soil rich in microorganisms was employed as the test medium, leveraging its high microbial activity to accelerate hydrogel decomposition. Based on prior findings, the EG and SG protein hydrogel groups demonstrated superior swelling properties, structural stability, and lower production costs. Therefore, the degradation study specifically focused on a comparing these two promising protein-based hydrogels with a petroleum-based gel (commercial water bead). As shown in Fig. 7A, the extent of biodegradation over a 21-day period was monitored. After 7 days, the biodegradability of the EG group reached 98.02%, significantly higher than that of the SG group (78.60%) and the water bead (petroleum-based gel) (0.00%) (*p* < 0.05). By day 14, the SG group reached a comparable biodegradation level to EG (98.03%), both significantly higher than that of the water bead (4.43%) (*p* < 0.05). At day 21, EG and SG groups had almost completely degraded, while the water bead showed limited biodegradation at only 11.51%. The biodegradation process in soil burial tests typically follows 3 stages (Thombare et al., 2018; Sarmah and Karak, 2020). In the first stage, microbial activity initiates the breakdown of the hydrogel network and accelerates decay. As water content increases, oxygen diffusion becomes restricted, creating anaerobic conditions that slow down microbial growth and degradation. In the final stage, water-induced fragmentation of the hydrogel matrix facilitates microbial access to smaller polymer chains, promoting complete degradation. Due to its looser structure, the EG group disintegrated more rapidly in the early phase, resulting in faster degradation. In contrast, the SG hydrogels with its denser and more robust structure exhibited all 3 phases distinctly. Its compact structure initially resisted water-induced softening, thereby delaying biodegradation. As shown in Fig. 7B, mold growth (red-circled region) was evident in soil with EG and SG samples after 7 days, in contrast to the soil with water beads, which remained visually unaffected. These observations confirm that the protein hydrogels not only disintegrated but also were actively biodegraded by microorganisms, rather than merely dissolving into invisible fragments. Furthermore, as a protein-rich material, the hydrogel provided nutrients that supported microbial proliferation, enhancing its environmental bioavailability and minimizing post-disposal ecological impact. In addition, the slower degradation rate of the SG group, attributed to its compact structure, may offer potential applications in prolonged water retention for agricultural irrigation.

## Conclusion

**Fig. 8:**
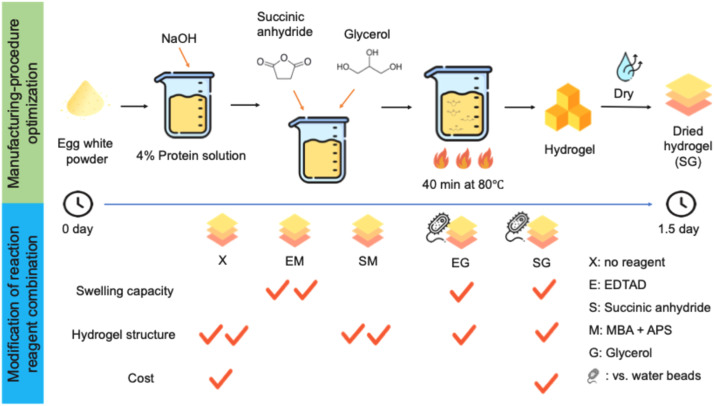
Schematic of the optimized manufacturing process and modified reaction reagent combination for egg-white based hydrogels.

This study successfully developed a protein-based hydrogel utilizing abnormal eggs, offering a cost-effective and sustainable biogenic material for waste valorization, as illustrated in Fig. 8. Initially, the hydrogel preparation method was optimized based on previous literature. Omitting the several steps, second freeze-drying, heating and grinding, helps to reduce the production time from 4 days to just 1.5 days. Subsequently, various acylating and cross-linking agents were incorporated to enhance the functional properties of the hydrogel. Comparative evaluation of hydrogel formulations revealed that the unmodified control, SM, and EM groups exhibited either poor water absorption or insufficient gel structure, rendering them unsuitable for further application. Among the remaining formulations, the SG and EG groups showed superior structural integrity, water retention capacity, and biodegradability, while maintaining relatively low production costs. Nevertheless, their biocompatibility and bioavailability profiles still require validation through rigorous safety testing. Looking forward, this work opens new avenues for transforming food industry by-products into value-added biomaterials. In practical use as food absorbent pads, materials are exposed to high ionic strength, protein- and lipid-rich meat exudates, mechanical compression, and refrigerated storage, all of which may influence gel swelling, liquid retention, and structural stability. Therefore, the current results should be interpreted as baseline performance rather than a direct simulation of industrial conditions. Further validation under real food processing conditions is needed before practical implementation. These findings suggest that future studies may focus on systematically evaluating the performance of SG or EG hydrogels under conditions that more closely mimic real food packaging environments, including saline solutions and model meat exudates with varying ionic strengths, as well as under mechanical compression at refrigerated temperatures. Such investigations will provide a more comprehensive understanding of the long-term absorbency, liquid retention, and structural durability of protein-based hydrogels for food absorbent pad applications.

## CRediT authorship contribution statement

**Yu-Shan Chang:** Data curation, Formal analysis, Investigation, Methodology, Visualization, Writing – original draft. **Jr-Wei Chen:** Validation, Resources, Methodology, Investigation. **Sheng-Yao Wang:** Validation, Methodology. **Yi-Chen Chen:** Writing – review & editing, Validation, Supervision, Resources, Project administration, Investigation, Funding acquisition, Data curation, Conceptualization.

## Disclosures

The authors declare that they have no known competing financial interests or personal relationships that could have appeared to influence the work reported in this paper.

## Appendix Supplementary materials

Image, application 1

## Footnotes

Supplementary material associated with this article can be found, in the online version, at doi:10.1016/j.psj.2026.106472.
