# Supplementary material for: Valorization of egg-white byproducts into biodegradable hydrogels: Processing optimization and functional properties
Source: Poult Sci. 2026 Jan 18;105(3):106472. doi: 10.1016/j.psj.2026.106472 (PMC12870771; doi:10.1016/j.psj.2026.106472)
Supplement: Supplementary file 1 [file mmc1.docx]

**Suppl. Table 1. Experimental groups and procedures for the optimized manufacturing of egg-white based hydrogels.**

| **A. Effects of different protein concentration on properties of absorbent hydrogels** | | | | | | | |
| --- | --- | --- | --- | --- | --- | --- | --- |
| Group | Egg white powder (g) | Water  (g) | EDTAD  (g) | MBA  (g) | APS  (g) | 60℃ heating  for 30min | Grinding |
| 3% | 1.698 | 28.302 | 0.180 | 0.117 | 0.056 | × | × |
| 4% | 2.264 | 27.736 | 0.240 | 0.156 | 0.075 | × | × |
| 5% | 2.830 | 27.170 | 0.300 | 0.195 | 0.093 | × | × |
| **B. Effect of heating at 60℃ or not on properties of absorbent hydrogels** | | | | | | | |
| Group | Egg white powder (g) | Water  (g) | EDTAD  (g) | MBA  (g) | APS  (g) | 60℃ heating  for 30min | Grinding |
| 4% ∆ (heated) | 2.264 | 27.736 | 0.240 | 0.156 | 0.075 | ✓ | × |
| 4% | 2.264 | 27.736 | 0.240 | 0.156 | 0.075 | × | × |
| **C. Effects of grinding or not on properties of absorbent hydrogels** | | | | | | | |
| Group | Egg white powder (g) | Water  (g) | EDTAD  (g) | MBA  (g) | APS  (g) | 60℃ heating  for 30min | Grinding |
| Powder | 2.264 | 27.736 | 0.240 | 0.156 | 0.075 | × | ✓ |
| Dried gel | 2.264 | 27.736 | 0.240 | 0.156 | 0.075 | × | × |

1. The dosages of these chemical reagents were following the optimal concentrations by Rathna et al. (2004) and Pourjavadi et al., (2006): 0.2g EDTAD/g protein, 0.015M MBA (0.13g/g protein), and 2.86 mM APS (0.062g/g protein).

2. Protein concentration in egg white powder: 0.53 g/g egg white powder.

7

**Suppl. Table 2. Experimental groups and treatments involving different reaction reagents and their combinations in the manufacture of egg-white based hydrogels.**

| **A. Effects of different succinic anhydride (SA) concentration on properties of absorbent hydrogels** | | | | | | | | | | | | | | |
| --- | --- | --- | --- | --- | --- | --- | --- | --- | --- | --- | --- | --- | --- | --- |
| Group | Egg white powder (g) | | Water (g) | | | SA (g) | | | | MBA (g) | | | APS (g) | |
| Control-1 | 2.264 | | 27.736 | | | - | | | | 0.156 | | | 0.075 | |
| 0.05 SA | 2.264 | | 27.736 | | | 0.060 | | | | 0.156 | | | 0.075 | |
| 0.1 SA | 2.264 | | 27.736 | | | 0.120 | | | | 0.156 | | | 0.075 | |
| 0.15 SA | 2.264 | | 27.736 | | | 0.180 | | | | 0.156 | | | 0.075 | |
| 0.2 SA | 2.264 | | 27.736 | | | 0.240 | | | | 0.156 | | | 0.075 | |
| 0.4 SA | 2.264 | | 27.736 | | | 0.480 | | | | 0.156 | | | 0.075 | |
| **B. Effects of different glycerol (G) concentration properties of absorbent hydrogels** | | | | | | | | | | | | | | |
| Group | Egg white powder (g) | | | Water (g) | | | | EDTAD (g) | | | | G (g) | | |
| Control-2 | 2.264 | | | 27.736 | | | | 0.240 | | | | - | | |
| 0.3 G | 2.264 | | | 27.736 | | | | 0.240 | | | | 0.360 | | |
| 0.4 G | 2.264 | | | 27.736 | | | | 0.240 | | | | 0.480 | | |
| 0.5 G | 2.264 | | | 27.736 | | | | 0.240 | | | | 0.600 | | |
| 0.6 G | 2.264 | | | 27.736 | | | | 0.240 | | | | 0.720 | | |
| **C. Effects of different combinations of acylation and cross-linking reagents on properties of absorbent hydrogels** | | | | | | | | | | | | | | |
| Group | Egg white powder (g) | Water (g) | | | EDTAD (g) | | SA (g) | | MBA (g) | | APS (g) | | | G (g) |
| X | 2.264 | 27.736 | | | - | | - | | - | | - | | | - |
| EM | 2.264 | 27.736 | | | 0.240 | | - | | 0.156 | | 0.075 | | | - |
| SM | 2.264 | 27.736 | | | - | | 0.180 | | 0.156 | | 0.075 | | | - |
| SG | 2.264 | 27.736 | | | - | | 0.180 | | - | | - | | | 0.600 |
| EG | 2.264 | 27.736 | | | 0.240 | | - | | - | | - | | | 0.600 |

1. The SA concentration was calculated based on protein concentration. Ex: 0.15SA: 2.264 × 0.53 × 0.5 = 0.180 [Egg white powder × (g protein per g powder) × SA concentration = SA dosage (g)].

2. The G concentration was calculated based on protein concentration. Ex: 0.5G: 2.264 × 0.53 × 0.5 = 0.60 [Egg white powder × (g protein per g powder) × G concentration = G dosage (g)].

3. Protein concentration in egg white powder: 0.53 g/g egg white powder.


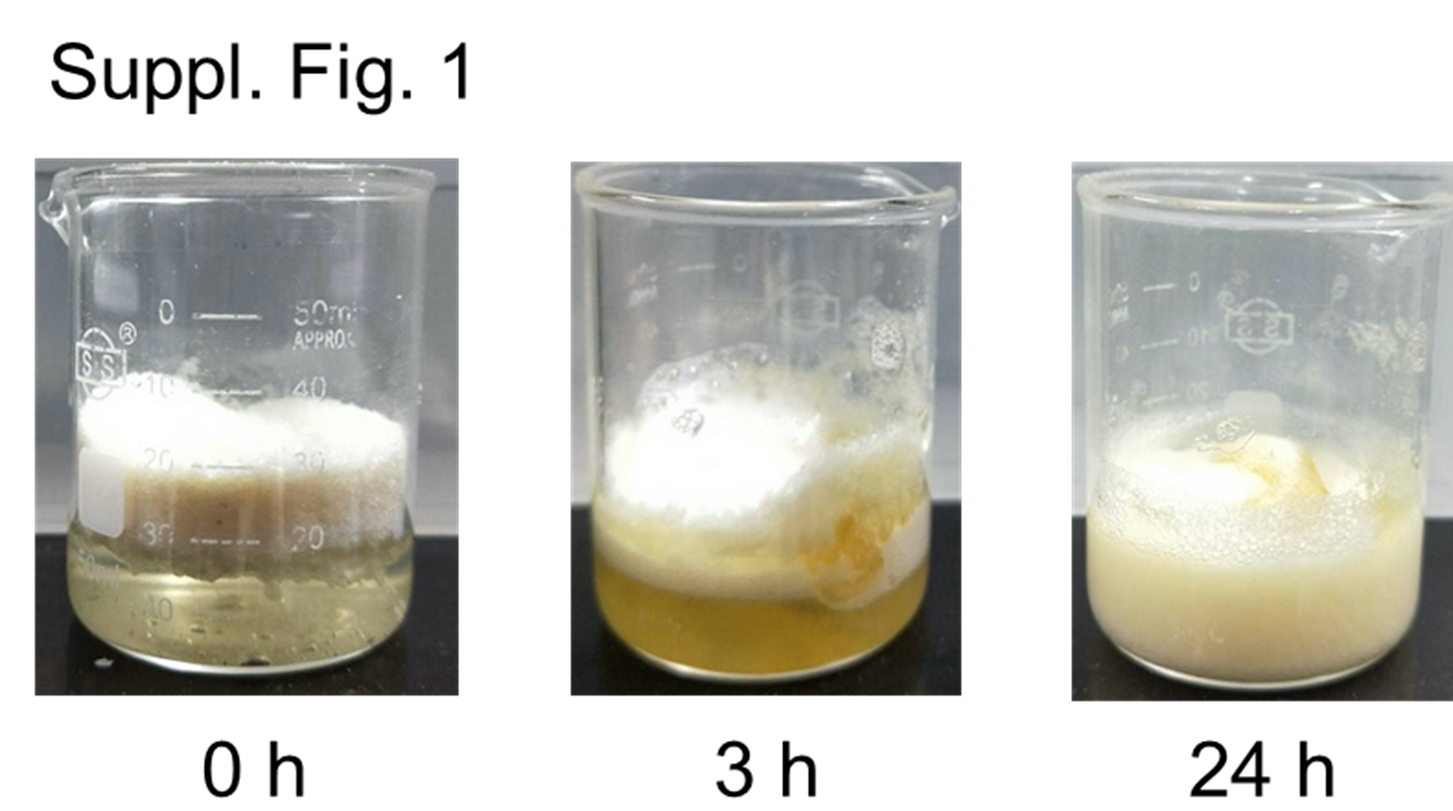


**Suppl. Figure 1. Appearance of the modified egg-white powder after the second round of lyophilization, following 0, 3, and 24 hours of dissolution.**
